# Assessing Depression Related Severity and Functional Impairment: The Overall Depression Severity and Impairment Scale (ODSIS)

**DOI:** 10.1371/journal.pone.0122969

**Published:** 2015-04-13

**Authors:** Masaya Ito, Kate H. Bentley, Yuki Oe, Shun Nakajima, Hiroko Fujisato, Noriko Kato, Mitsuhiro Miyamae, Ayako Kanie, Masaru Horikoshi, David H. Barlow

**Affiliations:** 1 National Center for Cognitive Behavior Therapy and Research, National Center of Neurology and Psychiatry, Kodaira, Japan; 2 Boston University, Boston, Massachusetts, United States of America; 3 National Institute of Mental Health, National Center of Neurology and Psychiatry, Kodaira, Japan; 4 Tokyo Medical University, Tokyo, Japan; 5 University of Tsukuba, Tsukuba, Japan; 6 National Center of Neurology and Psychiatry, Kodaira, Japan; National Center of Neurology and Psychiatry, JAPAN

## Abstract

**Background:**

The Overall Depression Severity and Impairment Scale (ODSIS) is a brief, five-item measure for assessing the frequency and intensity of depressive symptoms, as well as functional impairments in pleasurable activities, work or school, and interpersonal relationships due to depression. Although this scale is expected to be useful in various psychiatric and mental health settings, the reliability, validity, and interpretability have not yet been fully examined. This study was designed to examine the reliability, factorial, convergent, and discriminant validity of a Japanese version of the ODSIS, as well as its ability to distinguish between individuals with and without a major depressive disorder diagnosis.

**Methods:**

From a pool of registrants at an internet survey company, 2830 non-clinical and clinical participants were selected randomly (619 with major depressive disorder, 619 with panic disorder, 576 with social anxiety disorder, 645 with obsessive–compulsive disorder, and 371 non-clinical panelists). Participants were asked to respond to the ODSIS and conventional measures of depression, functional impairment, anxiety, neuroticism, satisfaction with life, and emotion regulation.

**Results:**

Exploratory and confirmatory factor analysis of three split subsamples indicated the unidimensional factor structure of ODSIS. Multi-group confirmatory factor analysis showed invariance of factor loadings between non-clinical and clinical subsamples. The ODSIS also showed excellent internal consistency and test–retest intraclass correlation coefficients. Convergence and discriminance of the ODSIS with various measures were in line with our expectations. Receiver operating characteristic curve analyses showed that the ODSIS was able to detect a major depressive syndrome accurately.

**Conclusions:**

This study supports the reliability and validity of ODSIS in a non-western population, which can be interpreted as demonstrating cross-cultural validity.

## Introduction

Depression is a common, debilitating mental health problem. There is a clear need to assess depression in various clinical and research settings, including psychiatry, primary care, community mental health, epidemiological studies, and session-by-session monitoring during outpatient treatment. To date, numerous self-report measures for depression have been developed and validated. For example, the Beck Depression Inventory-II (BDI-II) [1], Center for Epidemiological Studies-Depression (CES-D) [2], Patient Health Questionnaire-9 (PHQ-9) [3], Quick Inventory of Depression Scale (QIDS) [4], and Kessler Psychological Distress Scale (K6) [5] are all widely used in research and clinical practice [6,7]. Because each scale has different strengths and limitations in terms of reliability, validity, interpretability, responsiveness, and feasibility, it is important to consider the psychometric properties of each measure during use in clinical practice or for research purposes [6,7].

Most of these commonly used depression scales assess the frequency of somatic or cognitive–affective symptoms related to depression. For example, items of the PHQ-9 and QIDS were derived from diagnostic criteria for major depressive disorder (MDD): diminished interest or pleasure, depressed mood, insomnia or hypersomnia, fatigue or loss of energy, decrease or increase in appetite, feelings of unworthiness or excessive or inappropriate guilt, difficulties with concentration, psychomotor agitation or retardation, and thoughts of death or suicidal ideation [3,8]. The BDI-II and CES-D assess the other additional symptoms related to depression. The assumption of these scales is that somatic and cognitive–affective symptoms reflect the clinical importance or severity of depression.

However, functional impairment due to depressive symptoms is also of clinical importance [9]. Indeed, the Diagnostic and Statistical Manual-5 [10] includes functional impairment or significant distress resulting from depression as an indispensable criterion of MDD and other depressive disorder diagnoses (e.g., persistent depressive disorder). During treatment for any type of depression (including the range of depressive disorders and subclinical depressive symptoms), it is important to monitor how depression affects a patient’s daily life and interpersonal relationships, rather than only addressing the severity or frequency of somatic and cognitive–affective symptoms.

The Overall Depression Severity and Impairment Scale (ODSIS) was developed to address these important aspects of depression, namely, symptom severity and functional impairment as a single, underlying construct [9]. The ODSIS was adapted directly from the Overall Anxiety Severity and Impairment Scale (OASIS) [11,12]. As with the OASIS, the ODSIS assesses not only frequency or intensity of symptoms, but also functional impairment due to depression. Its applicability to the range of depressive disorders and subclinical symptoms are key differences from more conventional, longer depressive measures such as BDI-II, PHQ-9, QIDS, or CES-D [9]. In terms of feasibility, the ODSIS items can be answered using either detailed descriptions of each anchor or abbreviated anchors. The present study used an abbreviated version of the ODSIS, which provides one-word descriptions for each response option, as compared to the detailed descriptions of each anchor included in the original instrument [9]. The abbreviated ODSIS takes approximately 0 to 2 minutes to answer. Because of its brevity, the ODSIS is expected to be extremely well-suited for use in various settings such as epidemiological research, routine clinical monitoring, and primary care. For example, a recently developed, transdiagnostic cognitive-behavior treatment protocol uses the original ODSIS for session-by-session monitoring of depressive symptoms [13].

To date, one validation study using clinic outpatients (*n* = 100), university students (*n* = 566), and community adults (*n* = 189) in the United States reported on the reliability and validity of the original version of ODSIS [9]. Results showed excellent internal consistency (Cronbach’s alpha = .91–.94) and a unidimensional factor structure. Convergent and discriminant validity were demonstrated by correlations in expected directions with established measures of depression, anxiety, and temperament. In terms of classification accuracy, an ODSIS score of 8 or higher was able to accurately detect those individuals who met criteria for a depressive disorder diagnosis in the outpatient sample.

This initial validation study, however, had some limitations. First, the sample size, especially for outpatients diagnosed with depression (*n* = 24), was relatively small. Second, although a notable strength of the five-item ODSIS is its brevity, this validation study used the original version of ODSIS, which provides full descriptions (i.e., 1–3 brief sentences) for each response option. The original ODSIS is three times longer than the abbreviated version used in the present study, which, as we have noted, contains significantly shorter response options (original ODSIS 642 words, abbreviated ODSIS 199 words). To the authors’ knowledge, the reliability and validity of this abbreviated, and potentially more feasible, version of ODSIS have yet to be examined. Third, test–retest reliability, another important aspect of reliability, was not examined in the original investigation, and thus noted as an important direction for future research on the ODSIS [9]. Fourth, the factorial validity between non-clinical and clinical populations has not yet been investigated. Fifth, although the previous study showed that the ODSIS was well able detect clinical depressive disorders, the authors provided only one cut-off point, which may limit the interpretability of the full range of ODSIS scores. Sixth, cross-cultural validity of ODSIS has not been demonstrated in any investigations to date.

The current study was designed to elucidate these unknown aspects of reliability, validity, and interpretability of the abbreviated version of ODSIS using a large sample from Japanese non-clinical and clinical populations. First, we examined the factorial validity with exploratory and confirmatory factor analytic methods. Second, the reliability of ODSIS was examined in terms of both internal consistency and test–retest reliability. Third, the convergent and discriminant validity were examined in terms of correlations with related and unrelated constructs. Fourth, we examined the performance of the ODSIS in detecting a major depressive syndrome status. We calculated the Stratified Stratum Likelihood Ratio (SSLR) to obtain information for interpreting the range of ODSIS scores.

## Methods

### Participants and Procedures

This study is derived from a larger project for examining emotion and psychopathology in Japanese clinical and non-clinical populations. A validation study on the OASIS, which used data from the same sample, has been published elsewhere [14]. For this project, we conducted a web-based survey by following the electronic research methodology guidelines [14]. Participants 18 years old or older were recruited from registrants with Macromill Inc., the largest internet marketing research company in Japan. Among their 1,095,443 registrants, 389,265 are registered as “disease panelists.” Disease panelists are defined by an annual self-report of current or past diagnosis of a disease. We recruited participants with both current and past diagnoses because it may reduce the stratum bias [15]. Of the non-disease and disease panelists (9561 MDD, 3370 panic disorder (PD), 19,511 social anxiety disorder (SAD), and 971 obsessive–compulsive disorder (OCD) panelists at the time of February 2013), 2830 participants were selected randomly based on age, gender, and living area in each panelist group for the present study. These anonymous participants answered the Time 1 questionnaire packet (619 for MDD, 619 for PD, 576 for SAD, 645 for OCD, and 371 for non-disorder panelists; female, 1547; male, 1283; mean age, 42.44; *SD*, 10.39; range, 19–79) in January or May 2014. A subset of the January participants also completed the Time 2 survey during March 2014 (total 1050, 205 each for PD, SAD, OCD, MDD, and non-disorder panelists). Measures were administered in random order across individual administrations within both Time 1 and Time 2 surveys. Details about study participants have been described elsewhere [14].

### Ethics Statement

The institutional review board (IRB) at the National Center of Neurology and Psychiatry approved the ethical and scientific validity of this study (approval number: A2013-022). Prior to responding to study questionnaires, participants were asked to read the explanation of the study and ethical considerations. It was stated that participation in this study is voluntary and no disadvantages will result from not participating the study. We considered selecting the “agree” option as providing informed consent to participate. Only participants who selected “agree” could proceed to the study questionnaires. The IRB approved these procedures for obtaining informed consent in this anonymous survey-based study.

### Measures

#### Diagnostic status

At Time 1, we assessed current diagnostic status (i.e., presence of MDD, PD, SAD, OCD, and “other mental disorders” at the time of survey). Specifically, the item used to assess MDD was “Are you currently diagnosed as having Major Depressive Disorder and being treated for the problem in a medical setting?” Similar questions were used for PD, SAD, OCD, and other mental disorders (e.g., “Are you currently diagnosed as having panic disorder and being treated for the problem in a medical setting?”). We also asked the participants whether they had any experience using medical services such as psychiatric and psychosomatic clinics because of their psychological problem or difficulties.

#### Overall Depression Severity and Impairment Scale (ODSIS)—abbreviated version

The ODSIS was developed to assess depression in the following domains: frequency (Item 1), intensity (Item 2), functional impairment in pleasurable activity (Item 3), work or school (Item 4), and interpersonal relationships (Item 5) [9]. Items of ODSIS are scored on a five-point Likert scale of 0–4. As previously noted, in the current study, we used an abbreviated version of the ODSIS. In comparison to the detailed description of each anchor point included in the original version of ODSIS [9], the abbreviated version uses one Japanese word for each anchor (e.g., None). Details about the anchors and back-translation procedures for the ODSIS into Japanese are provided as Supporting Information (S1 Text).

#### Measures for convergent and discriminant validity

To examine the convergent validity of ODSIS, we used the Patient Health Questionnaire (PHQ-9) [3,16], the Center of Epidemiologic Studies Depression Scale (CES-D) [2], the Kessler Psychological Distress Scale (K6) [5], the Sheehan Disability Scale (SDS) [17], the State-Trait Anxiety Inventory—Trait (STAI) [18], the Generalized Anxiety Disorder 7-item scale (GAD-7) [19], the short-form revised Eysenck Personality Questionnaire—Neuroticism subscale (EPQR-N) [20], and the Satisfaction With Life Scale (SWLS) [21]. The Emotion Regulation Questionnaire—suppression subscale (SUP) [22] was also used to examine the discriminant validity. Information related to the reliability and validity of convergent and discriminant measures is included as Supporting Information (S1 Text).

### Statistical analyses

There were no missing data for this study because we used a web-based survey in which responses were required. Total ODSIS scores in clinical and non-clinical groups were calculated using summing responses to the five ODSIS items. Clinical groups were categorized based on their responses to the items assessing diagnoses of MDD, PD, SAD, OCD, and other mental disorders. A non-clinical group without a clinical history was comprised of individuals with no positive answers to these items and no self-reported history of using medical services to address psychological problems. If participants answered positively to the history of using medical services, but negatively to all items assessing current diagnostic status, then they were categorized as the non-clinical group with a clinical history. Participants who endorsed “other mental disorders” were excluded from all statistical analyses except for the descriptive statistics of the ODSIS. Correlations of ODSIS scores with sex, age, household income, personal income, living area, marital status (0, not married; 1, married), presence/absence of children (0, no child; 1, have child), and the number of psychiatric disorders were also examined.

We randomly split the total sample (*n* = 2784) into three subsamples to examine the factorial validity of ODSIS. Subsamples 1 and 2 (*n* = 886, 895 respectively) were used for two independent exploratory factor analyses (EFA). Subsample 3 (*n* = 903) was used for confirmatory factor analysis (CFA). Model fit was examined by inspecting goodness-of-fit indices, modification indices (M.I.), and correlation residuals [23–26]. Suggested criteria for good fit included non-significance of the chi-square test (χ^2^), standardized root-mean residual (SRMR) = < 0.08, Tucker–Lewis indices (TLI) > = .95, comparative fitness index (CFI) > = .95, and root-mean-square-error of approximation (RMSEA) = < .06 [25]. Following these indices, we modified the model for the CFA for subsample 3. Then, we conducted multi-group CFA using the total sample to assess the invariance of factor loadings in non-clinical and clinical subsamples. The aim of these analyses was to ascertain whether the model of interest provides good fit to the data even when invariance restrictions between non-clinical and clinical subsamples are imposed.

The reliability of the ODSIS was examined by calculating Cronbach’s alpha and test–retest intraclass correlation coefficients (ICC) within a two-month interval. Existing guidelines suggest that the ICC should be higher than .75 or .80 in order to indicate acceptable test-retest reliability [27]. Correlation analyses were conducted to evaluate the convergent and discriminant validity of ODSIS. In terms of convergent validity, the ODSIS was expected to be strongly correlated with the PHQ-9, CES-D, K6, and SDS, and to be moderately correlated with the STAI, GAD-7, EPQR-N, and SWLS. With regard to discriminant validity, we expected that the ODSIS would not be correlated with the SUP because the suppression is consistently uncorrelated with depression among Japanese people [28].

A ROC analysis was then conducted to examine the ODSIS’ ability to detect a major depressive syndrome status. We used validated criteria from the PHQ-9 [16] to define our categorical variable for major depressive syndrome status. Specifically, we classified participants as meeting criteria for major depressive syndrome status if they endorsed at least five of the nine PHQ-9 symptoms as being present on at least “more than half the days” (> = 2) in the past two weeks, with one of those symptoms being either depressed mood or diminished interest or pleasure. Any positive endorsement (> = 1) of items related to suicidal ideation was counted one major depressive symptom. The areas under the curve (AUC) were calculated to examine how accurately the ODSIS detects individuals’ major depressive syndrome status. We also calculated the SSLR of the ODSIS, which is a ratio of two likelihoods: one shows the test result in question among those with the target disorder and the other one shows the same test result among those without disorder. The SSLR approach presents some strength over the traditional threshold approach [29–31]. First, the SSLR approach provides multiple types of information for each stratum (i.e. range of the scores), whereas the traditional approach provides only one cut-off point. This SSLR information can be used to assist in the interpretation of scale scores [6]. Second, there is less spectrum bias in the SSLR approach as compared to a traditional threshold approach that has only one cut-off point. In SSLR analyses, both extremely severe and mild cases can be distributed in any of the strata, which results in less influence on the calculation of the likelihood ratio. If the SSLR is higher than 10, then the targeted disorder is highly probable. If it is lower than 0.1, then the targeted disorder is ruled out [31]. Instruments for which the SSLR is within the range of 0.1–2.0 are regarded as having no significance in detecting the target status.

IBM AMOS 22.0 was used for CFA. A spreadsheet (Excel, Microsoft Corp., Nagoya City University Evidenced-based Psychiatry Center http://www.ebpcenter.com) was used for calculating SSLR and its 95% confidence interval (CI). This spreadsheet has been used in several studies to date [29,32]. SPSS software (SPSS Statistics 22.0; IBM Corp.) was used for other statistical analyses.

## Results

### Preliminary analyses

The mean ODSIS score for the total sample was 6.51 (*SD* = 6.25). Participants were divided based on responses to items regarding diagnostic status. If participants did not respond positively to either of the items regarding current diagnostic status and clinical history, they were categorized as non-clinical group without a clinical history. A significant difference was found between non-clinical (*M* = 3.67, *SD* = 4.87) and clinical subsamples (*M* = 8.68, *SD* = 6.32; *t* (2681.83) = 23.20, *p* < .000, η^2^ = .158). Table 1 presents ODSIS scores in each subgroup. Of note, clinical groups with multiple diagnoses and non-clinical group with clinical history tended to score higher on other well-validated measures of depression (e.g., PHQ-9, CES-D; see S1 Table in Supporting Information). ODSIS scores were not significantly correlated with sex or living area (|*rs|* < .03, *n*.*s*.). Weak correlations were observed between ODSIS scores and age, marital status, presence/absence of children, household income, and personal income (*r* = -.15,-.22,-.19,-.20, and-.10, respectively, *p* < .000). ODSIS scores were positively correlated with the number of psychiatric disorders among clinical participants (*r* = .49, *p* < .000).

**Table 1:** ODSIS scores in non-clinical and clinical samples.

	*n*	*Mean*	*SD*	*SE*	95% CI
**Clinical groups**					
MDD only	406	9.14	6.01	0.30	8.56–9.73
PD only	198	3.66	4.66	0.33	3.00–4.31
SAD only	116	4.75	5.54	0.51	3.73–5.77
OCD only	66	4.56	5.32	0.65	3.25–5.87
MDD & PD	127	10.50	5.82	0.52	9.47–11.52
MDD & SAD	95	10.80	5.86	0.60	9.61–11.99
MDD & OCD	100	10.47	5.35	0.53	9.41–11.53
PD & SAD	39	7.28	6.01	0.96	5.33–9.23
PD & OCD	20	6.50	5.74	1.28	3.82–9.18
SAD & OCD	18	9.78	5.46	1.29	7.06–12.49
MDD, PD, & SAD	51	11.12	5.96	0.83	9.44–12.79
MDD, PD, & OCD	52	11.56	5.91	0.82	9.91–13.20
MDD, SAD, & OCD	55	12.16	5.17	0.70	10.76–13.56
PD, SAD, & OCD	22	7.59	6.76	1.44	4.59–10.59
MDD, PD, SAD, & OCD	156	12.28	5.57	0.45	11.39–13.16
Other mental disorders	146	6.19	5.77	0.48	5.25–7.14
**Non-clinical groups**					
Without clinical history	654	2.87	4.40	0.17	2.53–3.21
With clinical history	509	4.70	5.25	0.23	4.24–5.15
**Total**	2830	6.49	6.23	0.12	6.26–6.72

MDD, major depressive disorder; PD, panic disorder; SAD, social anxiety disorder; OCD, obsessive–compulsive disorder

### Factorial validity

The EFA for subsample 1 using principal factor solution without rotation explained 84.79% of variance in ODSIS scores. Eigenvalues for the first and second factor were, respectively, 4.24 and 0.28. Factor loadings on the first factor ranged from .89–.94. The same EFA procedure was conducted on subsample 2; the principal factor explained 84.74% of variance with an eigenvalue of 4.24. Factor loadings on the first factor were .88–.94. Together, these analyses support the unidimensional factor structure of ODSIS.

A CFA was conducted using subsample 3 to examine the unidimensional model’s goodness of fit to the data. Fit indices for the model were adequate: χ^2^ (5) = 406.93, *p* < .000, SRMR = 0.032, RMSEA = .299, 90% CI = .274–.323, TLI = .852, CFI = .926, AIC = 426.925. Modification indices indicated one point of strain between the error terms of item 1 and item 3 improves the goodness of fit (M.I. = 193.77). In addition, the correlation residual was .188 between these two items. Therefore, we added covariance between the error terms of item 1 (frequency of depression) and item 3 (impairment in pleasurable activities). We hypothesized that these two items had correlated error variance because items 1 and 3 assess the frequency of depression and impairment in pleasurable activity because of depression, respectively, using the same anchor point (“*None*” to “*All the time*”). Fit indices for this modified model were improved: χ^2^ (4) = 175.53, *p* < .000, SRMR = 0.020, RMSEA = .218, 90% CI = .191–.246, TLI = .921, CFI = .968, AIC = 197.52. The correlation between error terms of items 1 and 3 was significant (*r* = .54, *p* < .000). Modification indices and correlation residuals showed no need for additional improvement of the model.

Next, we conducted multi-group CFAs by dividing the samples into non-clinical (*n* = 1163) and clinical subsamples (*n* = 1521). Following the CFA explained above, we compared four models to assess the equivalence of estimation between non-clinical and clinical subsamples. Model 1 assumed no equivalence for the estimation. Model 2 assumed that factor loadings are the same across groups. Model 3 additionally assumed the same variance for the latent factor. Model 4 further assumed that all estimations including covariances between error terms and variances of the error terms are the same. As Table 2 shows, the chi-square test of Model 1 and Model 2 was not significant even in this large sample (χ^2^(4) = 12.93, *p* = .012), suggesting the equivalence of factor loadings between the non-clinical and clinical subsamples. The other nested model comparisons showed statistically significant differences between Model 1 and Models 3 and 4. Therefore, we regarded Model 2 as providing the best fit to the abbreviated ODSIS. In this model, standardized factor loadings were .84–.93 in the non-clinical subsample and .87–.94 in the clinical subsample. Correlations between the error terms of items 1 and 3 were significant in both non-clinical and clinical samples (*r* = .50 and .53, respectively, *ps* < .000).

**Table 2:** Goodness of fit indices for four types of equivalence restriction on a one-factor model with error theory of the ODSIS.

Model	χ^2^	df	SRMR	RMSEA	(90%CI)	TLI	CFI	AIC	Δχ^2^	df	*p*
Model 1	293.45	8	.015	.115	.104–.127	.955	.982	337.45	–	–	
Model 2	306.38	12	.017	.096	.087–.105	.969	.981	342.38	12.93	4	.012
Model 3	395.20	13	.038	.105	.096–.114	.963	.976	429.20	101.75	5	.000
Model 4	655.04	19	.018	.112	.104–.119	.957	.959	677.04	361.59	11	.000

Model 1 imposed no equivalence restriction.

Model 2 had equivalence restriction on factor loadings.

Model 3 had equivalence restriction on factor loadings and variance of latent factor.

Model 4 included equivalence restriction on factor loadings, variance of latent factor, covariances and variances of error terms.

### Reliability

Cronbach’s alpha was .96 for both non-clinical and clinical subsamples. The test–retest ICC with two month intervals was .75 (*n* = 602, *p* < .000) in the non-clinical subsample and.73 (*n* = 386, *p* < .000) in the clinical subsample.

### Convergent and discriminant validity

As shown in Table 3, the ODSIS was correlated strongly with functional impairment (i.e., SDS), measures of depression (i.e., CES-D, PHQ-9, K6) and one measure of anxiety (i.e., GAD-7). Scores on the ODSIS were moderately correlated with another measure of anxiety (i.e., STAI), the EPQR-N and SWLS, and were not significantly with SUP. These results in clinical and non-clinical populations were generally in line with our expectations.

**Table 3:** Correlations of ODSIS with measures for convergent and discriminant measures.

	ODSIS	CES-D	PHQ-9	K6	SDS	STAI	GAD-7	EPQR-N	SWLS	SUP
ODSIS	-	**.75**	**.74**	**.70**	**.84**	**.60**	**.72**	**.50**	**-.37**	.03
CES-D	**.77**	-	**.83**	**.78**	**.72**	**.76**	**.77**	**.60**	**-.55**	.04
PHQ-9	**.78**	**.83**	-	**.77**	**.70**	**.71**	**.82**	**.59**	**-.48**	.01
K6	**.71**	**.79**	**.79**	-	**.66**	**.81**	**.77**	**.62**	**-.51**	.03
SDS	**.81**	**.71**	**.71**	**.67**	-	**.60**	**.69**	**.48**	**-.36**	.02
STAI	**.63**	**.75**	**.72**	**.81**	**.59**	-	**.71**	**.71**	**-.64**	-.01
GAD-7	**.70**	**.76**	**.83**	**.78**	**.68**	**.71**	-	**.63**	**-.44**	-.02
EPQR-N	**.43**	**.51**	**.52**	**.58**	**.41**	**.61**	**.56**	-	**-.43**	-.03
SWLS	**-.34**	**-.46**	**-.40**	**-.44**	**-.32**	**-.58**	**-.34**	**-.28**	-	.03
SUP	.00	.01	.00	-.04	-.03	-.05	-.07	-.03	.09	-

Correlations above the diagonal correspond to the non-clinical sample and those below the diagonal correspond to the clinical sample.

Correlations in bold denote statistical significance of *p* < .000

ODSIS, Overall Depression Severity and Impairment Scale; CES-D, Center of Epidemiologic Studies Depression Scale; PHQ-9, Patient Health Questionnaire; K6, Kessler Psychological Distress scale, SDS, Sheehan Disability Scale; STAI, State-Trait Anxiety Inventory—Trait; GAD-7, Generalized Anxiety Disorder 7-item scale; EPQR-N, Short-form revised Eysenck Personality Questionnaire neuroticism subscale; SWLS, Satisfaction With Life Scale; SUP, Emotion Regulation Questionnaire suppression subscale

### Performance of the ODSIS in detecting a major depressive syndrome

**Fig 1:**
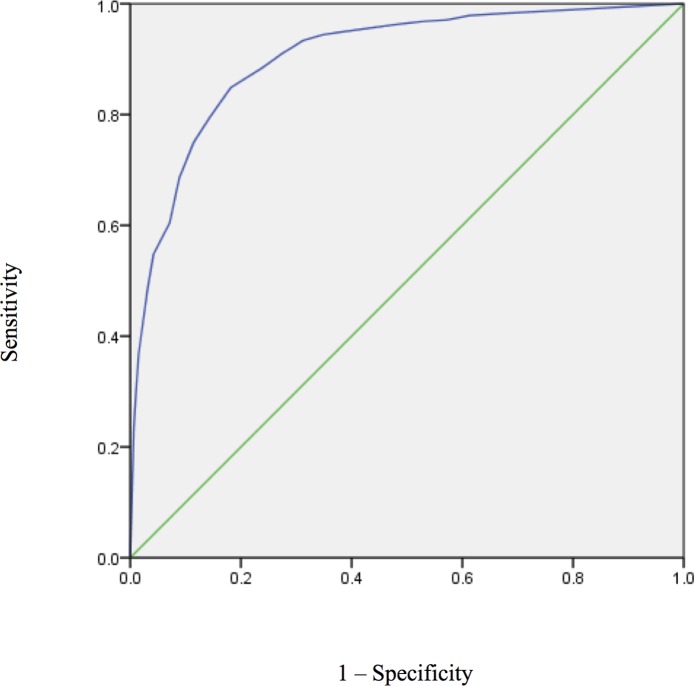
ROC curve for ODSIS scores to detect the presence of major depressive syndrome.

The AUC of ODSIS for detecting the presence of a major depressive syndrome was .904 (95% CI = .887–.920; Fig 1). Table 4 shows the SSLR of the ODSIS score stratum. Using the traditional threshold approach, the optimal cut-off score from the perspective of the balance of sensitivity and specificity was 11 or higher. The sensitivity, specificity, and correct classification for a cut-score of 11 were .85, .81, and 82.3%, respectively.

**Table 4:** Stratum-Specific Likelihood Ratio of ODSIS scores in detecting major depressive syndrome status.

Scores	Positive	Negative	SSLR	95% CI
0–5	19	1420	0.08	(0.05–0.13)
6–8	15	256	0.36	(0.22–0.59)
9–11	43	300	0.88	(0.65–1.19)
12–14	72	168	2.63	(2.04–3.39)
15–17	89	129	4.23	(3.31–5.42)
18–20	138	35	24.2	(17.02–34.42)

## Discussion

### Main findings

This study was designed to examine the psychometric properties of the ODSIS using large clinical and non-clinical populations in Japan. The ODSIS was found to have excellent internal consistency and good test–retest reliability. A unidimensional factor structure was confirmed. Correlations with various measures indicated convergent and discriminant validity. The ODSIS performed well in detecting the major depressive syndrome status. Information about the likelihood of meeting criteria for major depressive syndrome status in each stratum (i.e., score range) was obtained.

### Factorial validity

This study not only replicated the unidimensional factor structure of the ODSIS shown in its initial validation [9], but also demonstrated the invariance of factor loadings between non-clinical and clinical populations. Results from this study show that aspects of functional impairment and frequency and intensity of depression symptoms are explained by the same latent factor. Given that most existing depression scales do not include items assessing functional impairment due to depression, these results are particularly noteworthy. The demonstrated invariance of factor loadings also enables clinicians to interpret total ODSIS scores without considering differences across individual items between non-clinical and clinical populations. Future research using structural equation modeling might choose to use partial modification of the unidimensional structure (i.e., covariance of error terms for item 1 and 3) suggested by this study.

### Validity in detecting diagnostic status of major depressive syndrome

The ODSIS performed well in detecting a diagnosis of major depressive syndrome. Traditional ROC curve analysis showed that a score of 11 or higher on the ODSIS was optimal in terms of balancing sensitivity and specificity. This cut-score met the criteria suggested by Matthey and Petrovski (sensitivity of .70 and specificity of .80)　[33]. The correct classification was 82%, which is the same value for the optimal cut-off score of 8 in the U.S. [9]. However, the SSLR for the stratum of 12–14 was only 2.63, which means that the respondent whose score was 12–14 is only approximately three times more likely to have an major depressive syndrome status than what would be expected by chance. Therefore, clinicians may prefer to use a more probable score range such as 15 or more to more accurately identify those individuals with a probable major depressive syndrome status.

Although this study indicated 11 or higher as the optimal cutoff for probable major depressive syndrome, the previous validation study using a U.S. sample suggested a cut-score of 8 or higher for determining a clinical depressive disorder diagnosis [9]. This difference might reflect one or more of the following differences between the previous and present study: sample size (*n* = 100 vs. 2684), characteristics of participants (e.g., treatment-seeking outpatients only vs. a broader sample consisting of both treatment-seeking and non-clinical individuals, American vs. Japanese), diagnostic assessment procedures (independent semi-structured diagnostic interview conducted prior to answering the ODSIS vs. simultaneously administered self-report items), or number of depressive disorders assessed (any clinical depressive disorder vs. major depressive syndrome only). Additionally, we included “past diagnosis” panelists to minimize the stratum bias [15], whereas the earlier study used only current mood disorders. This difference in cut-off scores might also be a function of differences in mean ODSIS scores (5.50 for U.S. clinical sample vs. 6.51 for the Japanese clinical sample examined here). Considering these important factors, it may not be altogether surprising that we found a higher cutoff score using a very large sample of subjects with, overall, more severe symptoms in this study; however, future research is needed to replicate these findings. These results also might be consistent with the fact that the prevalence of depressive disorders is lower in Japan [34]. The possibility exists that even if the level of depressive symptomatology is high, an individual may not be meet major depressive syndrome status.

### Reliability

As reported in U.S. sample, the internal consistency was also excellent among Japanese non-clinical and clinical populations. Reliability was further supported by the test–retest stability. ICCs (.73–.75) were sufficient, particularly for a measure assessing depressive symptoms that are expected to change over time. These results indicate that 73–75% of variance in all ODSIS scores is attributable to variance in the underlying construct. The remaining 25–27% is attributable to error. Given that depression is prone to change depending the circumstances surrounding the depression (e.g., time, life event, treatment), the observed ICCs of .73–.75 are conceptually reasonable. Well-established depression measures such as BDI and CES-D also tend to show moderate test–retest stability [35]. One limitation, however, is that we cannot determine whether these results are attributable to reliability of the ODSIS or to participants’ depression stability at the two-months interval.

### Convergent and discriminant validity

Convergent and discriminant validity was supported by the magnitude of correlations of ODSIS scores with validated measures of depression, anxiety, and related variables. The ODSIS had strong correlations with the SDS, CES-D, PHQ-9, K6, and GAD-7. One noteworthy finding was the strong correlation between ODSIS and overall functional impairment assessed by SDS. This finding supports the construct validity of the ODSIS as a measure of functional impairment [9]. The ODSIS also evidenced correlations of at least moderate magnitude with measures of anxiety such as STAI. It is particularly noteworthy that other depression measures (i.e., CES-D and PHQ-9) showed slightly stronger correlation with the STAI and GAD-7. These results might imply that the ODSIS distinguishes between depression and anxiety better than other conventional measures of depression. These results may also reflect the unique character of ODSIS as a measure of functional impairment, rather than the presence of symptoms associated with a particularly disorder such as the CES-D, PHQ-9, STAI, and GAD-7. Correlations with EPQR-N, SWLS, and SUP were consistent with our expectations, showing convergent and discriminant validity.

### Limitations

This study has several limitations. First, participants were limited to the panelist pool of this one large internet marketing company. Participants also seemed to have lower income in comparison to results of the Japanese Census. In addition, these participants might be biased, with greater likelihood of including high internet users and strong willingness to participate in marketing research. In regard to the depressed population captured by the present study, patients with more severe depression may be less likely to register themselves as panelists and respond to online questionnaire surveys. Thus, the clinical sample used in the present study may not reflect the most severely depressed individuals. These limitations must be considered in combination with the strengths of using web-based survey methods; for example, we were able to recruit participants from all over Japan with a broad range of ages for the present investigation. Second, our definition of clinical and non-clinical populations was dependent on single self-report items only. Thus, we were unable to assess the accuracy of the responses by comparing them with data gleaned from a validated, clinician-rated diagnostic tool. Participants’ potential misunderstandings of their own diagnoses might increased the number of false positives in clinical sample. Third, the outcome variable for our ROC curve analysis was not administered independently from the ODSIS, and, perhaps more importantly, we were unable to use a validated, structured clinical interview to determine participants’ diagnostic status. Although the operational definition used to indicate major depressive syndrome has been partially validated in several prior studies [17], such a definition itself is relatively loose in comparison to DSM diagnosis and, as such, may have resulted in diagnostic inflation (i.e., PHQ-9 criteria for major depressive syndrome only requires “more than half the days” as opposed to DSM-IV criteria for major depressive disorder, which requires “nearly every day”).

Future research must use established measures with independent assessment to assess diagnostic status. Fourth, although the ODSIS is applicable to the full range of depressive disorders and subclinical symptoms, we did not include other depressive disorders such as persistent depressive disorder or bipolar disorders. Therefore, the validity of the ODSIS shown in this study is limited to MDD.

## Conclusion

Despite these limitations, this large-scale study demonstrated the invariance of factor loadings between non-clinical and clinical populations, strong correlations of the ODSIS with a measure of functional impairment and widely used depression scales, and provided useful information for interpreting total ODSIS scores. These results also support the cross-cultural validity of ODSIS. Because of its brevity and feasibility, it is expected that this scale will be well-suited for various clinical and research settings, particularly those in which it might not be feasible for respondents to complete lengthier depression measures.

## Supporting Information

S1 Dataset (XLSX)

S1 Table CES-D and PHQ-9 scores in non-clinical and clinical group. (DOCX)

S2 Table Hierarchical regression analysis of diagnostic status in predicting ODSIS score. (DOCX)

S1 Text Description of measures. (DOCX)
