# Supplementary material for: Assessing Depression Related Severity and Functional Impairment: The Overall Depression Severity and Impairment Scale (ODSIS)
Source: PLoS One. 2015 Apr 13;10(4):e0122969. doi: 10.1371/journal.pone.0122969 (PMC4395441; doi:10.1371/journal.pone.0122969)
Supplement: S1 Table — (DOCX) [file pone.0122969.s002.docx]

| **Table S1** |  |  |  |  |  |
| --- | --- | --- | --- | --- | --- |
| CES-D and PHQ-9 scores in non-clinical and clinical group | | | | | |
|  |  | CES-D | | PHQ-9 | |
|  | *n* | *Mean* | *SD* | *Mean* | *SD* |
| **Clinical groups** |  |  |  |  |  |
| MDD only | 406 | 26.54 | 13.40 | 12.42 | 7.57 |
| PD only | 198 | 19.07 | 11.80 | 8.02 | 6.48 |
| SAD only | 116 | 20.44 | 12.37 | 9.27 | 6.49 |
| OCD only | 66 | 21.15 | 12.12 | 10.29 | 6.65 |
| MDD & PD | 127 | 30.18 | 13.26 | 14.54 | 7.09 |
| MDD & SAD | 95 | 31.74 | 12.71 | 15.56 | 7.10 |
| MDD & OCD | 100 | 28.83 | 13.39 | 14.55 | 7.38 |
| PD & SAD | 39 | 29.10 | 14.16 | 12.87 | 6.86 |
| PD & OCD | 20 | 23.65 | 11.39 | 12.65 | 8.09 |
| SAD & OCD | 18 | 36.83 | 14.02 | 16.61 | 7.72 |
| MDD, PD, & SAD | 51 | 34.35 | 13.08 | 16.76 | 6.91 |
| MDD, PD, & OCD | 52 | 32.08 | 12.89 | 16.08 | 7.60 |
| MDD, SAD, & OCD | 55 | 35.29 | 11.29 | 17.20 | 6.32 |
| PD, SAD, & OCD | 22 | 27.00 | 11.85 | 12.27 | 6.39 |
| MDD, PD, SAD, & OCD | 156 | 33.99 | 13.27 | 17.12 | 7.26 |
| Other disorders | 146 | 24.20 | 12.62 | 10.92 | 7.06 |
| **Non-clinical groups** |  |  |  |  |  |
| Without clinical history | 654 | 15.75 | 10.62 | 6.00 | 6.18 |
| With clinical history | 509 | 19.71 | 12.02 | 8.20 | 6.59 |
| **Total** | 2830 | 23.12 | 13.69 | 10.42 | 7.74 |
