# Supplementary material for: Assessing Depression Related Severity and Functional Impairment: The Overall Depression Severity and Impairment Scale (ODSIS)
Source: PLoS One. 2015 Apr 13;10(4):e0122969. doi: 10.1371/journal.pone.0122969 (PMC4395441; doi:10.1371/journal.pone.0122969)
Supplement: S2 Table — (DOCX) [file pone.0122969.s003.docx]

**Supplementary Analyses**

**Contributions of specific diagnoses to predicting ODSIS scores**

A hierarchical regression analysis was conducted to examine the effects of specific disorders on ODSIS scores. Data from a single self-report item assessing “diagnosis of MDD and treatment for the MDD” was entered on the first step. A self-reported diagnosis of and treatment for PD, SAD, and/or OCD were entered on the second step. Both step 1 and step 2 models were statistically significant (*F* (1, 2683) = 894.14, *p* < .000, *R* = .500, *R^2^* = .250, and *F* (4, 2683) = 254.26, *p* < .000, *R* = .525, *R^2^* = .274). The second model explained a significant proportion of incremental variance (Δ*R^2^* = .025, *p* < .000). As illustrated in Table S2, the diagnostic and treatment status of MDD contributed a 6.41-point increase to predicting total ODSIS scores.

| **Table S2** |  |  |  |
| --- | --- | --- | --- |
| Hierarchical regression analysis of diagnostic status in predicting ODSIS score | | | |
| Diagnosis | B | B SE | *β* |
| *Step 1* |  |  |  |
| MDD | 6.41 | .21 | .50*** |
|  |  |  |  |
| *Step 2* |  |  |  |
| PD | 0.60 | .26 | .04* |
| SAD | 1.56 | .28 | .10*** |
| OCD | 1.37 | .30 | .08*** |
| MDD, major depressive disorder; PD, panic disorder; SAD, social anxiety disorder; OCD, obsessive compulsive disorder.  **p* < .05, ****p* < .001 | | | |
