# Supplementary material for: Assessing Depression Related Severity and Functional Impairment: The Overall Depression Severity and Impairment Scale (ODSIS)
Source: PLoS One. 2015 Apr 13;10(4):e0122969. doi: 10.1371/journal.pone.0122969 (PMC4395441; doi:10.1371/journal.pone.0122969)
Supplement: S1 Text — (DOCX) [file pone.0122969.s004.docx]

**Methods**

**Measures**

**Overall Depression Severity and Impairment Scale (ODSIS) – abbreviated version.** The ODSIS was recently developed to assess depression in the following domains: frequency, intensity, functional impairment in pleasurable activities, work or school, and interpersonal relationships [1]. Items of ODSIS are scored on a five-point Likert scale of 0–4. Items 1 (frequency of depression) and 3 (functional impairment in pleasurable activity) require responses from “None” to “All the time.” Items 2 (intensity of depression), 4 (impairment in work or school), and 5 (impairment in interpersonal relationships) take responses from “None” to “Extreme.” Following recommended procedures [2, 3], standard back-translation methods were used to translate the ODSIS into Japanese. First, one author (MI), a Japanese clinical psychologist, translated the ODSIS forward into Japanese. Second, the translation was reviewed by a committee comprising five Japanese clinical psychologists (MH, YO, NK, HF, SN) and one psychiatrist (AK), all of whom assessed the accuracy of the Japanese translations and expressions. Third, the modified Japanese translation was back-translated independently by a bilingual clinical psychologist who did not know the purpose of this study. Fourth, the back-translated English version was again examined and partly modified by the committee described above. Fifth, two of the original developers (KHB, DHB) of the ODSIS checked the concordance of the meanings between original and back-translated items. Phrases were modified in response to the original developers. The bilingual clinical psychologist then back-translated the modified Japanese version. We repeated these procedures four times. Finally, the original developers confirmed the content-validity of the scale. Although we translated the original version of ODSIS, which is printed in the Japanese translation of Barlow et al. (2010) [4], we used the abbreviated version in this study. In comparison to the detailed description of each anchor point included in the original version of ODSIS, the abbreviated version uses one Japanese word for each anchor (e.g., None). For example, the abbreviated anchors for item 4 (“In the past week, how much did your depression interfere with your ability to do the things you needed to do at work, at school, or at home?”) are 0: None 1: Mild, 2: Moderate, 3: Severe, 4: Extreme, whereas item 4 on the full version of the ODSIS uses the following anchors: 0: None: No interference at work/home/school from depression, 1: Mild: My depression has caused some interference at work/home/school. Things are more  difficult, but everything that needs to be done is still getting done, 2: Moderate: My depression definitely interferes with tasks. Most things are still getting done, but  few things are being done as well as in the past, 3: Severe: My depression has really changed my ability to get things done. Some tasks are still being  done, but many things are not. My performance has definitely suffered, and 4: Extreme: My depression has become incapacitating. I am unable to complete tasks and have had  to leave school, have quit or been fired from my job, or have been unable to complete tasks at home and have faced consequences like bill collectors, eviction, etc.

**Patient Health Questionnaire (PHQ-9).** This questionnaire assesses severity of depression by asking about the frequency of depressive symptoms during the prior two weeks [5, 6]. Items are rated from 0 (Not at all) to 3 (Nearly every day). The PHQ-9, widely used in clinical settings, has been validated as a measure of depression among clinical and general populations [5, 7].

**Center of Epidemiologic Studies Depression Scale (CES-D).** The CES-D scale is used broadly in epidemiological studies to screen for depression [8, 9]. Items inquire about the frequency of depressive symptoms during the prior week, and are rated from 0 (Rarely or none of the time; less than 1 day) to 3 (Most or all the time; 5–7 days). The reliability and validity of the CES-D have been demonstrated in U.S. and Japanese populations [8, 9].

**Kessler Psychological Distress Scale (K6).** This scale assesses global psychological distress during the prior 30 days [10, 11]. Items are taken from well-known scales of anxiety and depression such as the STAI, Beck Depression Inventory, and Center of Epidemiologic Studies Depression Scale (CES-D), and are rated from 0 (None of the time) to 4 (All of the time). The K6 items represent anxiety (nervous, restless) and depressive (depressed, everything was an effort, and worthless) symptoms. The scale has shown good performance in detecting individuals who meet criteria for mood and anxiety disorders [11].

**Sheehan Disability Scale (SDS).** This scale assesses functional impairment in three domains: work/school, social, and family life [12, 13]. In this study, respondents were asked to rate their degree of impairment from 0 (Not at all) to 5 (Extremely). The reliability and validity among various populations have been reported in a number of prior investigations [14].

**State-Trait Anxiety Inventory – Trait (STAI).** This 20-item questionnaire assesses the usual tendency to experience anxiety symptoms [15, 16]. Items are answered on a Likert scale from 1 (Never) to 4 (Always). More than 800 reported studies have used this scale. Its reliability for administration to the Japanese population have also been demonstrated in a number of investigations to date [16, 17].

**Generalized Anxiety Disorder 7-item scale (GAD-7).** This scale assesses symptoms of generalized anxiety during two weeks [18, 19]. Items are scored on a scale from 0 (Not at all sure) to 3 (Nearly every day). Reliability, as well as factorial and diagnostic criterion validity, have been reported for clinical populations [19].

**Short-form revised Eysenck Personality Questionnaire – Neuroticism subscale (EPQR-N).** This measure assesses the personality traits of extraversion, neuroticism, and psychoticism [20, 21]. We used the neuroticism subscale, consisting of 12 items, scored on a two-point Likert scale. Previous reports have described the reliability of the neuroticism subscale [20, 21].

**Satisfaction With Life Scale (SWLS).** This five-item measure assesses the global cognitive judgments of satisfaction with one’s life [22, 23]. It has been well-validated in many studies to date [23, 24].

**Emotion Regulation Questionnaire-suppression subscale (SUP).** This scale assesses two aspects of emotion regulation: reappraisal and suppression [25, 26]. The suppression subscale consists of four items answered on a seven-point Likert scale. Reliability and validity have been demonstrated in both U.S. and Japanese populations [25, 26].

**References**

1. Bentley KH, Gallagher MW, Carl JR, Barlow DH (2014) Development and validation of the overall depression severity and impairment scale. Psychological Assessment 26: 815-830.

2. Beaton DE, Bombardier C, Guillemin F, Ferraz MB (2000) Guidelines for the process of cross-cultural adaptation of self-report measures. Spine 25: 3186-3191.

3. Mokkink LB, Terwee CB, Patrick DL, Alonso J, Stratford PW, et al. (2010) The COSMIN checklist for assessing the methodological quality of studies on measurement properties of health status measurement instruments: an international Delphi study. Quality of Life Research 19: 539-549.

4. Barlow DH, Ellard KK, Fairholme CP, Farchione TJ, Boisseau CL, et al. (2010) Unified protocol for transdiagnostic treatment of emotional disorders: Workbook. New York: Oxford University Press.

5. Kroenke K, Spitzer RL (2002) The PHQ-9: a new depression diagnostic and severity measure. Psychiatric Annals 32: 1-7.

6. Muramatsu K, Miyaoka H, Kamijima K, Muramatsu Y, Yoshida M, et al. (2007) The patient health questionnaire, Japanese version: validity according to the mini-international neuropsychiatric interview-plus. Psychological Reports 101: 952-960.

7. Martin A, Rief W, Klaiberg A, Braehler E (2006) Validity of the Brief Patient Health Questionnaire Mood Scale (PHQ-9) in the general population. General Hospital Psychiatry 28: 71-77.

8. Radloff LS (1977) The CES-D scale a self-report depression scale for research in the general population. Applied psychological measurement 1: 385-401.

9. Shima S, Kano T, Kitamura T (1985) New self-report scale for depression. Seishin-Igaku 27: 717-723.

10. Furukawa TA, Kawakami N, Saitoh M, Ono Y, Nakane Y, et al. (2008) The performance of the Japanese version of the K6 and K10 in the World Mental Health Survey Japan. International Journal of Methods in Psychiatric Research 17: 152-158.

11. Kessler RC, Andrews G, Colpe LJ, Hiripi E, Mroczek DK, et al. (2002) Short screening scales to monitor population prevalences and trends in non-specific psychological distress. Psychological Medicine 32: 959-976.

12. Sheehan D, Harnett-Sheehan K, Raj B (1996) The measurement of disability. International Clinical Psychopharmacology 11: 89-95.

13. Yoshida T, Ohtsubo T, Tsuchida H, Wada Y, Ueshima K, et al. (2004) Development of Sheehan Disability Scale (SDISS) and its reliability and validity. Rinsyo-Seishin-Yakuri 7: 1645-1653.

14. Leon AC, Shear MK, Portera L, Klerman GL (1992) Assessing impairment in patients with panic disorder: the Sheehan Disability Scale. Social Psychiatry and Psychiatric Epidemiology 27: 78-82.

15. Spielberger CD, Gorsuch RL, Lushene RE (1970) Manual for the state-trait anxiety inventory. Palo Alto: Consulting Psychologists Press.

16. Shimizu H, Imae K (1981) Development of the Japanese version of State-Trait Anxiety Inventory. The Japanese journal of educational psychology 29: 348-353.

17. Barnes LL, Harp D, Jung WS (2002) Reliability generalization of scores on the Spielberger state-trait anxiety inventory. Educational and Psychological Measurement 62: 603-618.

18. Muramatsu K, Miyaoka H, Ueshima K, Muramatsu Y, Fuse K, et al. (2010) Validation and utility of a japanese version of the GAD-7. Japanese Journal of Psychosomatic Medicine 50: 592.

19. Spitzer RL, Kroenke K, Williams JB, Lowe B (2006) A brief measure for assessing generalized anxiety disorder: the GAD-7. Archives of Internal Medicine 166: 1092-1097.

20. Eysenck SB, Eysenck HJ, Barrett P (1985) A revised version of the psychoticism scale. Personality and individual differences 6: 21-29.

21. Hosokawa T, Ohyama M (1993) Reliability and validity of a Japanese version of the short-form Eysenck Personality Questionnaire-Revised. Psychological reports 72: 823-832.

22. Diener E, Emmons RA, Larsen RJ, Griffin S (1985) The satisfaction with life scale. Journal of personality assessment 49: 71-75.

23. Sumino Z (1994) Development of the Japanese version of Satisfaction With Life Scale [SWLS]. Annual convention of the Japanese Association of Educational Psychology: 192.

24. Pavot W, Diener E (1993) Review of the satisfaction with life scale. Psychological assessment 5: 164-172.

25. Gross JJ, John OP (2003) Individual differences in two emotion regulation processes: implications for affect, relationships, and well-being. Journal of personality and social psychology 85: 348-362.

26. Yoshizu J, Sekiguchi R, Amemiya T (2013) Development of a Japanese version of Emotion Regulation Questionnaire. Japanese Journal of Research on Emotions 20: 56-62.
